# Apple Core Unveiled: Malignant Colonic Obstruction Revealing an Unknown Rectosigmoid Neoplasm With Foreign Body Impaction

**DOI:** 10.7759/cureus.51536

**Published:** 2024-01-02

**Authors:** Daniela Martins, Ricardo Vaz-Pereira, Cátia Ferreira, Pedro Costa, João Pinto-de-Sousa

**Affiliations:** 1 General Surgery, Centro Hospitalar de Trás-Os-Montes e Alto Douro, Vila Real, PRT; 2 General Surgery, Clinical Academic Centre Trás-Os-Montes e Alto Douro, Vila Real, PRT

**Keywords:** invasive mucinous adenocarcinoma, incidental colon cancer, impacted foreign body, colon cancer, colonic foreign body

## Abstract

This case report highlights a rare clinical scenario of a 46-year-old male presenting with constipation and fecaloid vomiting due to an impacted chicken bone within an unidentified rectosigmoid neoplasm, leading to acute malignant colonic obstruction. Emergent exploratory laparotomy revealed an impacted chicken bone lodged in a previously unknown rectosigmoid tumor. An anatomopathological examination revealed a mucinous adenocarcinoma with clear margins and one pericolic metastatic lymph node. The postoperative period was uneventful, and the patient was proposed for adjuvant chemotherapy. The abrupt onset of symptoms allowed for an early diagnosis, emphasizing the unexpected association between foreign body impaction and incidental malignant obstruction. This case underscores the complexity of managing foreign body ingestion in the gastrointestinal tract and emphasizes the crucial role of diagnostic imaging in surgical planning. Furthermore, it draws attention to the potential occurrence of colorectal cancer in younger individuals, emphasizing the necessity for clinical vigilance and screening strategies beyond conventional age recommendations.

## Introduction

The ingestion of foreign bodies (FBs) is a common reason for seeking care in the emergency ward. Approximately 80% of FBs pass through the gastrointestinal (GI) tract without causing any symptoms or complications, thereby requiring no external intervention. However, they may get lodged in narrow or angled sections of the intestinal lumen, such as the ileocecal valve, or areas narrowed by a neoplasm. Approximately 20% of cases necessitate endoscopic measures, and about 1% require surgical intervention [1,2].

The symptoms resulting from the ingestion of FB can range from no symptoms to nonspecific signs like abdominal discomfort. In more serious cases, they can lead to complications like intestinal obstruction, perforation, or fistulization. While FB ingestion is more common in children, it can also occur in adults. Clinicians should remain vigilant and consider this possibility [1,3].

Colorectal cancer (CRC) ranks as the third most common cancer globally, accounting for approximately 10% of all cancer cases and one of the leading causes of cancer-related death worldwide [4].

Despite active screening efforts, the incidence of obstructive colon cancer remains unchanged. Malignant colorectal intestinal obstruction, requiring surgical intervention or emergency procedures, is a recognized complication of advanced neoplasms and affects 10% to 18% of patients initially diagnosed with colon cancer [5,6].

This case report highlights the malignant narrowing of the sigmoid intestinal lumen caused by an unidentified rectosigmoid neoplasm, resulting in an impacted chicken bone that led to acute malignant colonic obstruction.

## Case presentation

A 46-year-old previously healthy male presented to the emergency department complaining of constipation and fecaloid vomits over the past two days. Initially, he started with constipation and alimentary vomits with a progressive clinical deterioration noticing abdominal distension and the absence of bowel movements or flatus. The patient also developed fecaloid vomits, which led him to seek medical care. The patient denied experiencing weight loss, anorexia, asthenia, or hematochezia over the past weeks or months.

**Figure 1:**
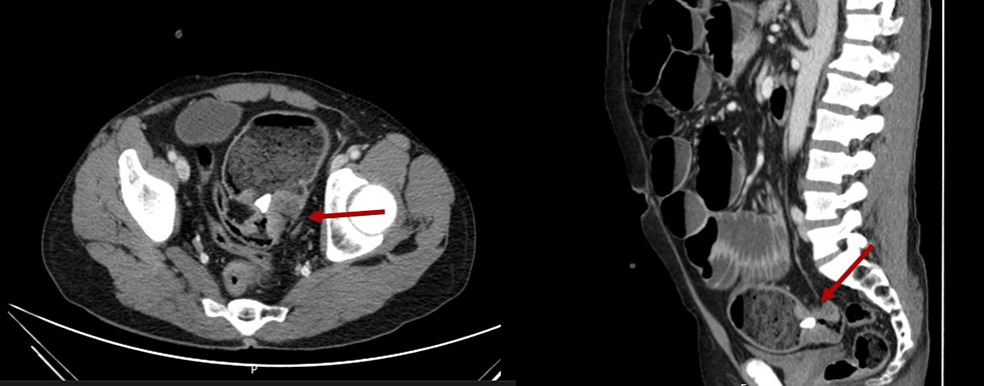
Abdominopelvic CT scan Findings of an apple core lesion situated at the rectosigmoid transition, containing a foreign body within, leading to complete colonic obstruction.

At admission to the emergency department, he exhibited normotension but was tachycardic (105 beats per minute). Upon examination, his abdomen appeared markedly distended and resonant, with diffuse mild tenderness and audible bowel sounds. Blood analyses showed a normal hemoglobin level (14.5 g/dL - normal for adult males ranging from 14 to 18 g/dl) and elevated inflammatory parameters with leukocytosis and neutrophilia (with normal values ranging from 4.500 to 11.000 WBCs per microliter (4.5 to 11.0 × 109/L)) and elevated C-reactive protein (with normal value of less than 0.3 mg/dL). Following an initial assessment involving fluid resuscitation, an abdominopelvic CT scan was performed, revealing air-fluid levels due to an apple core lesion located at the rectosigmoid transition, with a foreign body inside, indicating total bowel obstruction (Figure 1).

The surgical team initially opted for conservative management with nasogastric decompression, fluids, and an expectant approach with close observation. However, during the observation period, the patient’s clinical condition progressively deteriorated, maintaining tachycardia, with worsening abdominal distention, and increased pain with tenderness and guarding. These findings prompted the surgical team toward an emergent exploratory laparotomy. Rectosigmoidoscopy was not performed as it was not available at the institution at that time.

**Figure 2:**
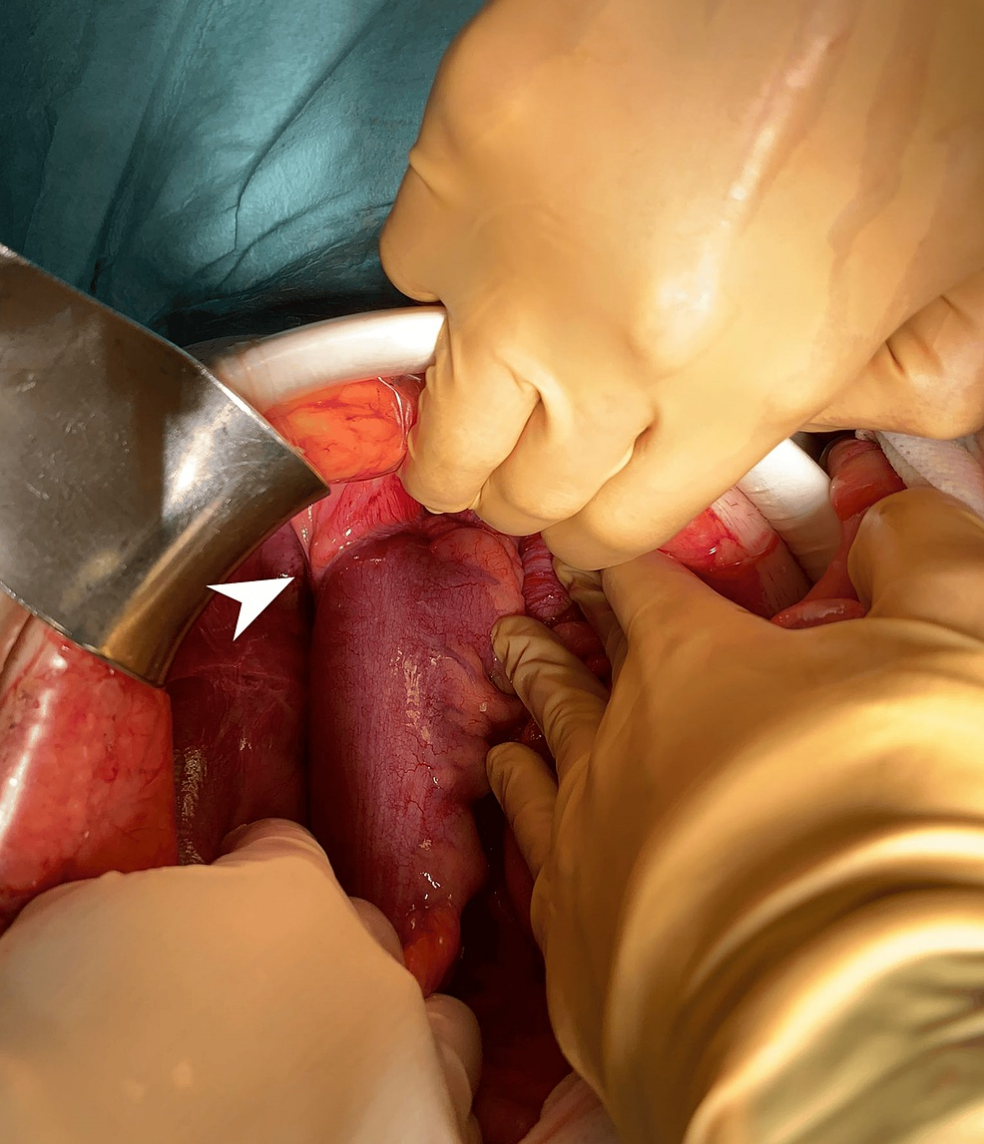
Sigmoid colon neoplasm invading parietal peritoneum (arrow)

**Figure 3:**
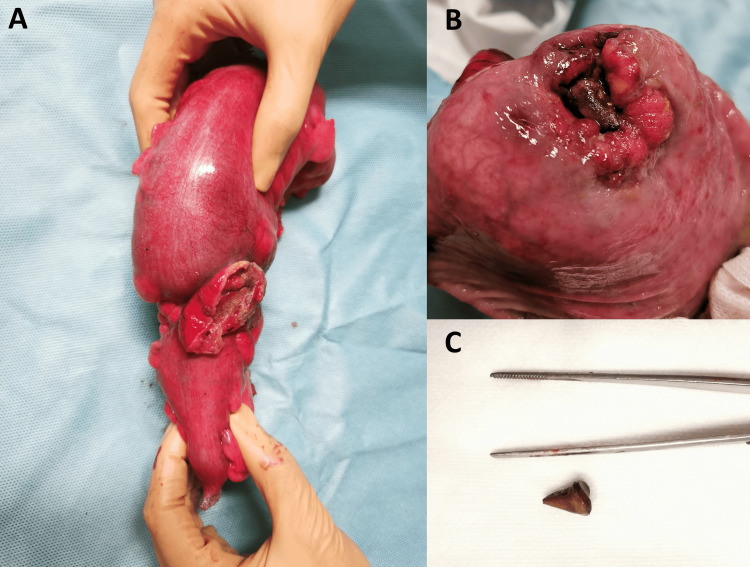
Surgical specimen and foreign body A: The surgical specimen displays tumoral expression on the serosa, along with a section of the parietal peritoneum that was resected with the specimen. B: Upon opening the surgical specimen, a tumoral mass and a foreign body are revealed. C: The foreign body is identified as a chicken bone.

Intraoperatively, remarkably distended bowel loops (small bowel and colon upstream of the obstruction point, at the rectosigmoid junction) were observed without signs of ischemia, alongside the identification of a sigmoid neoplasm infiltrating the parietal peritoneum (Figure 2). There were no associated lymphadenopathies, liver metastases, or peritoneal implants. Based on these findings, particularly the pronounced bowel distension, the surgical team opted for a Hartmann procedure instead of a primary anastomosis. The opening of the surgical specimen revealed a foreign body consistent with a chicken bone (2 x 3 cm), which was lodged in the tumor (Figure 3).

**Figure 4:**
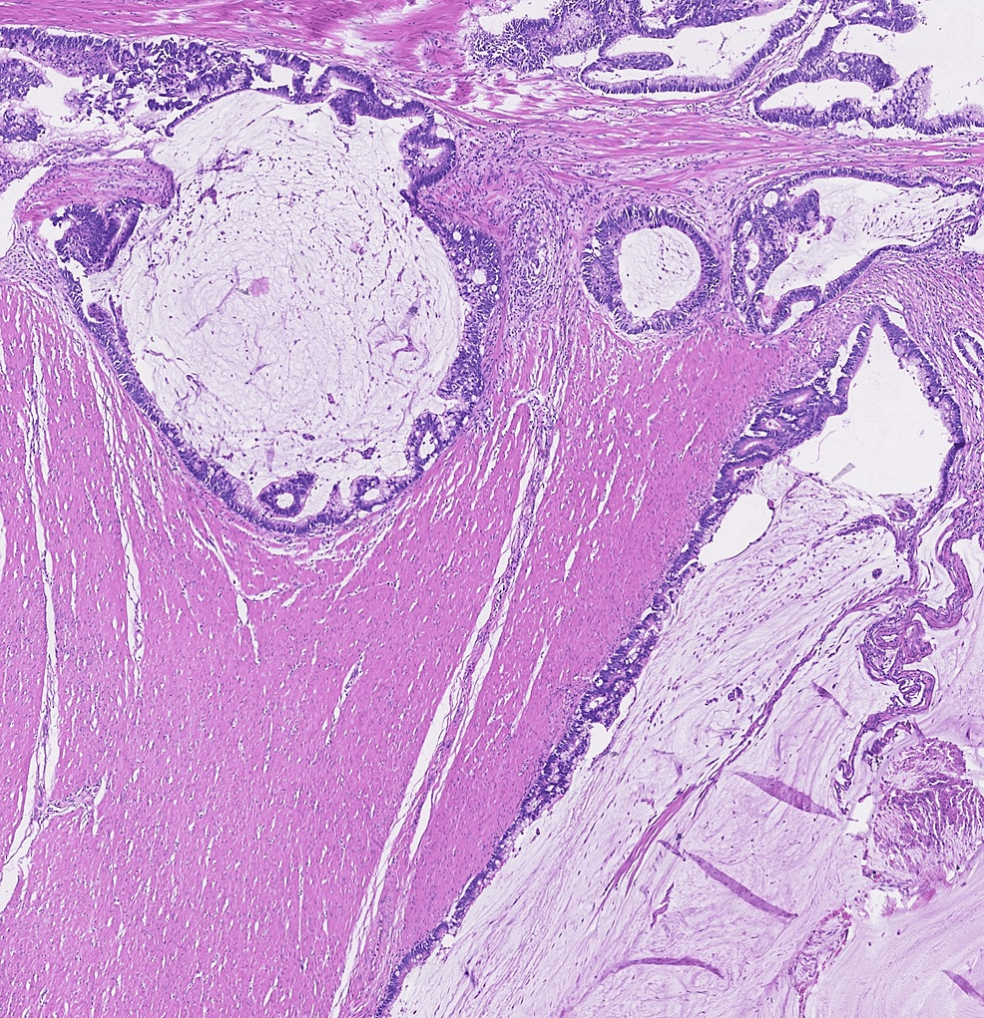
Histopathological findings Invasive mucinous adenocarcinoma of the sigmoid colon, with dissociation of the colonic wall by dilated mucus-producing neoplasia glands, with rupture and extravasation of mucus, reaching the peri-colic fat (hematoxylin and eosin staining, magnification 10x).

**Figure 5:**
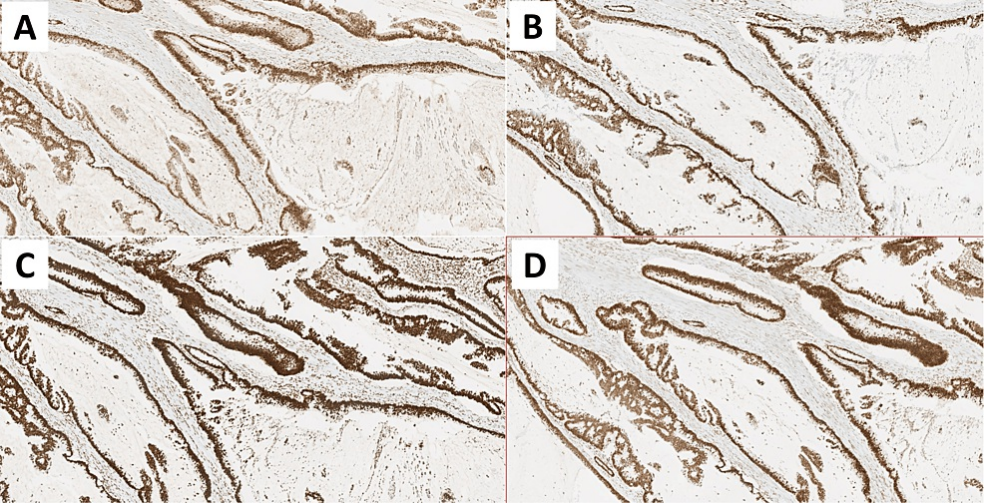
Histopathological findings Immunohistochemical staining with antibodies to MLH1 (A), PMS2 (B), MSH2 (C), and MSH6 (D), demonstrating nuclear expression of the four mismatch repair (MMR) proteins in the neoplastic cell population (magnification 5x).

Staging thoracoabdominopelvic CT scan revealed no distant metastasis and anatomopathological examination unveiled invasive mucinous adenocarcinoma (MAC) (Figure 4), with clear margins, corresponding to a stage III - T3N1aM0 - one metastatic ganglion in 11 excised per the American Joint Committee on Cancer Staging Manual (8th edition) Tumor Node Metastasis classification [7]. Immunohistochemistry assessment indicated a low likelihood of microsatellite instability (MSI) (Figure 5).

The postoperative period was uneventful, and the patient was discharged after eight days. After that, the multidisciplinary board decided on adjuvant chemotherapy.

## Discussion

FB ingestion may be incidental or intentional. In most cases, FBs pass through the GI tract spontaneously without causing complications, and patients remain asymptomatic. However, FB may become lodged at narrow or angled sections of the intestinal lumen, such as in the narrowing caused by a neoplasm. This can lead to unspecific symptoms or complications, including ulceration, bleeding, fistula or abscess formation, perforation, or GI obstruction [3,8].

In the presented case, the ingestion was accidental, and an obstruction, more specifically incidental malignant obstruction, occurred.

CRC is one of the most common cancers worldwide, with adenocarcinoma being the most prevalent histologic type accounting for 90% of the cases. Classical adenocarcinoma, MAC, and signet-ring cell carcinoma represent the three primary categories of adenocarcinoma [4,9].

MAC is a subtype of adenocarcinoma found in 10-20% of patients with CRC. It is characterized by a significant presence of mucinous components, constituting at least 50% of the tumor volume. These tumors are often diagnosed at more advanced stages compared to non-mucinous adenocarcinomas [9,10].

Concerning clinical pathology, MAC is more commonly found in the proximal colon than in the rectal or distal colon. Usually, mucinous colorectal adenocarcinomas are more frequent in females and younger patients [9].

MAC is linked to mutations affecting the Ras-Raf-MEK-ERK pathway (RAS/MAPK pathway) and a high occurrence of MSI. However, the factors contributing to the onset of MAC and their prognostic significance remain poorly understood. In the presented clinical case, there was no evidence of MSI [9].

Obstructive colon cancer itself presents as an independent high-risk factor for recurrence, and individuals with this condition often exhibit an advanced cancer stage with unfavorable prognostic factors. Furthermore, compared to patients undergoing elective surgery, those requiring emergency surgery for obstructive colon cancer experience poorer short-term and long-term oncologic outcomes [6].

In advanced cancers, malignant colorectal obstruction may be a serious complication and is usually insidious, presenting with non-specific GI symptoms, such as abdominal pain, vomiting, and abdominal distension [2]. In this case report, the patient presented with an acute onset of malignant colon obstruction, due to sudden impaction of a FB.

Abdominal X-ray and CT scan are the main imaging techniques for detecting FB in the GI tract, with CT scan being the more effective method. CT scans, readily available and highly effective, precisely locate obstructive cancer lesions with both high sensitivity and specificity in the abdominopelvic region [3,6]. In this clinical case, the CT scan played a crucial role in diagnosing the cause of the obstruction. This was due to the radiopaque nature of the FB and the revelation of a previously unknown tumor. The CT scan also played a key role in planning the surgical strategy. Unfortunately, rectosigmoidoscopy was not available, which could have been a viable approach for attempting to remove the FB [1].

## Conclusions

FBs found within the GI tract associated with a malignant colonic obstruction may pose a range of complex management challenges. Treatment and management should be based on the patient’s current health status and risk factors influencing short-term outcomes. In this case report, a young male was incidentally found to have a mucinous adenocarcinoma of the colon due to malignant obstruction and the presence of a foreign body. The abrupt symptoms, caused by FB impaction on neoplasm, facilitated an earlier diagnosis compared to the scenario if the neoplasm had naturally progressed to cause complete colonic obstruction.
